# Targeting Melanin Heterogeneity in Metastatic Melanoma: A Dual‐Tumour Mouse Melanoma Model

**DOI:** 10.1111/exd.70159

**Published:** 2025-09-02

**Authors:** Marine Delmas, Benjamin Chaussin, Nathan Harismendy, Aurore Dougé, Paul‐Olivier Rouzaire, Christopher Montemagno, Jérôme Durivault, Emmanuel Moreau, Elisabeth Miot‐Noirault, Mercedes Quintana, Sophie Besse, Michel D'Incan, Emmanuel Chautard, Elodie Jouberton, Jacques Rouanet

**Affiliations:** ^1^ UMR1240 INSERM, Université Clermont Auvergne Clermont‐Ferrand France; ^2^ Centre Jean PERRIN Service de Médecine Nucléaire Clermont‐Ferrand France; ^3^ Service d'Oncologie Médicale CHU Gabriel Montpied Clermont‐Ferrand France; ^4^ Laboratoire HLA CHU Gabriel Montpied Clermont‐Ferrand France; ^5^ Département de Biologie Médicale Centre Scientifique de Monaco Monaco France; ^6^ Service de Dermatologie et d'Oncodermatologie CHU Estaing Clermont‐Ferrand France; ^7^ Centre Jean PERRIN Service de Pathologie Clermont‐Ferrand France

**Keywords:** abscopal effect, immunotherapy, melanoma, targeted radionuclide therapy

## Abstract

The combination of melanin‐targeted radionuclide therapy (TRT) and immunotherapy offers potential in overcoming melanoma resistance to conventional therapies. Studying the potential abscopal effect induced by TRT is essential to evaluate such combination. We develop here a preclinical murine model comprising a target (pigmented) and non‐target (non‐pigmented) tumour to study the abscopal effect induced by melanin‐TRT in melanoma. Murine melanoma cell lines were tested: two pigmented (B16‐F10 and B16‐OVA) and one non‐pigmented (B16‐G4F), inoculated in C57BL/6 mice to assess pigmentation levels and immune infiltration. Heterogeneous tumour growth and repigmentation of the B16‐G4F tumour led us to develop a non‐pigmented cell line (B16‐OVAmTYR−/−) by tyrosinase invalidation using CRISPR/Cas9. A dual‐tumour model comprising the B16‐OVA tumour and the B16‐OVAmTYR tumour was evaluated in terms of tumour growth, pigmentation, and immune infiltrate. The B16‐OVA model displayed homogeneous tumour growth, pigmentation and high immune infiltrate (CD8+ T cells *p* < 0.001; CD4+ T cells *p* < 0.05, regulatory T cells *p* < 0.001). The new B16‐OVAmTYR−/− cell line ensured a consistent genetic background for comparative studies. The B16‐OVAmTYR−/− maintained a non‐pigmented phenotype without repigmentation (no melanin expression) and demonstrated similar tumour growth characteristics to its pigmented counterpart (DT = 2.4 ± 0.5 days). Establishing a dual‐tumour model using both B16‐OVA and B16‐OVAmTYR−/− cell lines enabled concurrent study of pigmented and non‐pigmented tumours in a single host, closely mirroring clinical scenarios of metastatic melanoma. We have successfully developed a new dual‐tumour pigmented and non‐pigmented mouse melanoma model mimicking clinical observations to study the abscopal effect in metastatic melanoma.

## Graphical abstract

The abscopal effect is known in external beam radiotherapy but unresolved in targeted radionuclide therapy (TRT) due to a lack of in vivo models. This study develops a B16‐OVA non‐pigmented melanoma cell line and a dual‐tumour model with pigmented and non‐pigmented melanoma to investigate the abscopal effect in melanin‐TRT.

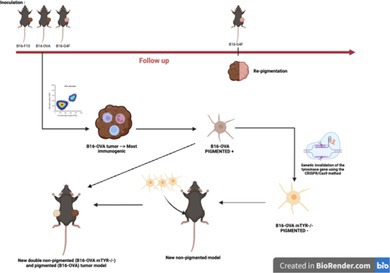

## 1 Introduction

Melanoma is a type of skin cancer that originates in melanocytes, skin cells responsible for melanin production [1]. Melanin is responsible for skin, hair and eye pigmentation and UV protection [2]. Early detection of melanoma is associated with a favourable prognosis, with a 5‐year survival rate of 99.4% for stage I to II disease. In contrast, late‐stage disease is still associated with a low survival rate (stage III: 68% and IV: 29.8%) [3, 4]. While the majority of melanoma subtypes are pigmented, about 5%–10% of cutaneous melanomas are amelanotic [1]. A key clinical feature of melanoma is its remarkable phenotypic plasticity, notably its ability to dedifferentiate and lose pigmentation. This intra‐patient heterogeneity complicates therapeutic decision‐making and is a major factor in treatment failure.

Since the 2010s, the introduction of BRAF and MEK inhibitors and immune checkpoint inhibitors targeting CTLA‐4 and PD‐1/PD‐L1 has revolutionised treatment strategies for melanoma [5]. There have been significant improvements in 5‐year survival rates with a median progression‐free survival of 11 months for BRAF + MEK inhibitors [6] and 11.5 months for anti‐CTLA‐4 + anti‐PD‐1 treatments [7]. However, only 68% of patients with BRAF‐mutated melanoma respond to BRAF + MEK inhibitors, with a median duration of response of 13.8 months [8]. Around 40%–55% of patients have immune checkpoint inhibitors (ICIs) innate resistance and 25% of them develop resistance during treatment [9]. These limitations highlight the need for new therapeutic strategies and for a better understanding of the mechanisms contributing to treatment response and failure.

Among the emerging concepts is the abscopal effect, a rare but intriguing immunologic phenomenon observed with radiotherapy [10]. It refers to the regression of non‐irradiated metastatic lesions following localised irradiation and is thought to result from radiation‐induced immunogenic cell death (ICD) and systemic activation of anti‐tumour immunity, particularly CD8^+^ T cells [11, 12].

Its potential to convert localised treatments into systemic therapies makes the abscopal effect a compelling field of investigation—particularly in tumours like melanoma, which can present with mixed lesions of varying pigmentation and immunogenicity.

Although initially described in external beam radiotherapy (EBRT), the potential of other radiotherapeutic modalities, such as Targeted Radionuclide Therapy (TRT), to induce abscopal effects remains largely unexplored.

TRT is an emerging and rapidly evolving approach in nuclear medicine, designed to selectively deliver radiopharmaceuticals to tumours [13]. The ionising radiation emitted mainly induces direct DNA damage in tumour cells and also modulates anti‐tumour immune responses [14]. In murine models of melanoma, β‐emitting TRT has been shown to trigger the release of damage‐associated molecular patterns (DAMPs) [15, 16], promote the recruitment of immune cells—including CD4^+^ and CD8^+^ T cells [17, 18], as well as NK cells [16]—and potentially enhance tumour immunogenicity. This dual mode of action opens the possibility that TRT might induce systemic immune responses capable of targeting distant, non‐irradiated lesions—a phenomenon known as the abscopal effect.

Despite its therapeutic potential, the abscopal effect remains poorly characterised, largely due to the scarcity of reliable and clinically relevant preclinical models. Melanoma, which often presents with both pigmented and non‐pigmented (dedifferentiated) metastases within the same patient, offers a unique context to investigate this phenomenon. However, most existing models fail to replicate such phenotypic heterogeneity.

In this context, we developed a novel dual‐tumour mouse model featuring one pigmented and one non‐pigmented melanoma lesion in the same animal. This model closely mirrors the clinical coexistence of phenotypically distinct metastatic sites and provides a robust platform to study spatially heterogeneous responses to therapy. Beyond TRT, this dual‐tumour system offers a valuable tool for the preclinical assessment of various therapeutic modalities in melanoma, including immune checkpoint inhibitors (ICIs) and targeted therapies, in a setting that more accurately reflects the clinical complexity of the disease.

## 2 Material & Methods

### 2.1 Cell Lines and Cell Culture

Murine B16‐F10 and B16‐OVA MO4 pigmented melanoma cell lines were purchased from ATCC Manassas USA and Sigma‐Aldrich Darmstadt Germany, respectively. B16‐G4F, obtained from Dr. Mireille Verdier (EA 3842, Université de Limoges), is a non‐pigmented B16‐F1‐derived cell line [19]. B16‐OVA were cultured in RPMI‐1640 culture medium supplemented with 10% foetal bovine serum, a solution of non‐essential amino acids (1X), 10 mM HEPES, 54 μM β‐mercaptoethanol, and 1 mg/mL geneticin. B16‐F10 and B16‐G4F were cultured in DMEM culture medium supplemented with 10% foetal bovine serum and with 4 μg/mL of gentamicin. Cell lines were maintained in an incubator at 37°C with 5% CO_2_. Cell culture reagents were purchased from Gibco, Fisher scientific, France, Strasbourg.

### 2.2 Genetic Disruption of Tyrosinase Using CRISPR‐Cas9

B16‐OVA cells were transfected using Xfect reagent (Takara) according to the manufacturer's instructions with pSpCas9(BB)‐2A‐GFP (PX458) plasmid (gift from Feng Zhang; Addgene plasmid #48138; http://n2t.net/addgene:48138; research resource identifier: Addgene_48 138) [20] containing clustered regularly interspaced short palindromic repeats (CRISPR) and guide RNA (gRNA).

Selected gRNA target regions of the mouse tyrosinase gene were designed using the ChopChop webtool [21]. The gRNA guides used in this study are CrmTyr_exon1 5′GTCTGCCTGAAAGCTGGCCGC 3′; CrmTyr_exon2#1 5'GGGACCACTATTACGTAATCC‐3′; CrmTyr_exon2#5 5′GTTATGCGATGGAACACCTGA. A Guanine was added to the 5′ end due to the transcription initiation requirement of a ‘G’ base for human U6 promoter.

GFP positive cells were sorted by single‐sorting FACS analysis (BD FACSMelody, Allschwil, Switzerland) at 24 h post‐transfection and seeded in 96‐well plates in their medium. Screening of clones via Western blot allowed the validation of knockouts (KO) and the selection of one KO for subsequent experiments, to minimise the clonal heterogeneity. The clones were cultured in B16‐OVA medium.

### 2.3 Doubling Time Measurement

B16‐OVA and B16‐OVAmTYR−/− cells were seeded in 24‐well plates at different concentrations (5000; 10 000; 15 000 and 20 000 cells per well). Cells were monitored in real time every 6 h for 72 h using Incucyte (Sartorius). Cell growth has an exponential function (*N = N0.e* ^*a.t*^), doubling time (DT) was calculated using the formula *DT = Ln2*/*a*, where “N” represents confluence of cells, “N0” represents confluence at time 0, “*a*” represents the slope of the exponential phase, and “*t*” the time (in hours).

### 2.4 Murine Models

Mice experiments were approved by the local Ethics committee of Clermont‐Ferrand and the French Ministry of Education and Research (Approval No. 2022050215457597). Melanoma cells were inoculated into 5‐week‐old female C57BL6‐J mice from Charles River (L'Arbresle, France). 2 × 10^5^ B16‐F10, B16‐OVA and B16‐G4F cells and 5 × 10^5^ B16‐OVAmTYR−/− in 100 μL PBS were subcutaneously (s.c) injected in the right flank for mono‐tumour models. For the double tumour model, B16‐OVA (pigmented) cells were injected into the right flank and B16‐OVAmTYR−/− (non‐pigmented) cells into the left flank the same day. Tumour volume and weight were measured three times a week by the same operator. Tumour volume was calculated from two dimensions using a calliper according to the formula *L × S* ^2^ */2*, where *L* and *S* are the largest and smallest diameters, respectively, expressed in millimetres. DT of tumour growth was calculated in the same way as for cell DT except “*t*” is expressed in days. After the sacrifice of animals at different tumour stages (250 and 1000 mm^3^), the tumours were separated into two parts: one for cytometry and the other for histological studies.

### 2.5 Analysis of Melanin Expression

Melanin presence was determined using Fontana Masson staining on histological sections using the manufacturer's protocol (ab150669, Abcam, Amsterdam, the Netherlands).

Total melanin amount was quantified using a spectrophotometry method on tumours stored at −80°C. The detailed protocol was already published [22].

An HPLC method was used to quantify eumelanin (EM) and pheomelanin (PM) in frozen tumours according to Rioux et al., in 2019 [23].

### 2.6 Western‐Blot Analysis

Western‐blot analysis was carried out as previously described [24]. Anti‐tyrosinase antibody (ab170905, 1/1000, Abcam, Amsterdam, the Netherlands) was used as the primary antibody, and anti‐rabbit IgG HRP (4050–05, 1/5000, Southern Biotech, Birmingham, USA) was used as the secondary antibody. Chemidoc Touch MP was used for revelation (Bio‐Rad, Paris, France).

### 2.7 Immunofluorescence

Immunofluorescence was performed as previously described [16]. The following primary antibodies were used: anti‐calreticulin (Abcam ab92516), anti‐HMGB1 (Invitrogen PA1‐16926), anti‐CD3 (Abcam ab5690), anti‐CD8a (Invitrogen 14–0808‐82). The following secondary antibodies were used: anti‐rabbit (Invitrogen A‐21247), anti‐rat (Invitrogen A‐11008), anti‐mouse (A21422). Slides were mounted using Fluoroshield with DAPI (Sigma Aldrich, USA). Histological images were acquired with the Axio Scan.Z1 slide scanner and the ZEISS Colibri 7 camera. Negative controls obtained without primary antibody are presented in Figure S1.

### 2.8 Flow Cytometry

Fresh tumours were dissociated using a mouse tumour dissociation kit (Miltenyi Biotec, Paris, France). Antibodies for flow cytometry were supplied by BD Biosciences, Grenoble, France. After filtration and washing, cells were incubated in staining buffer with a mixture of conjugated anti‐mouse antibodies for 15 min in the dark at 4°C. Selection was carried out among CD45+ cells. NK cells were identified as CD3‐/NKp46+, CD8+ T cells as CD45+/CD3+/CD8+, CD4+ T cells as CD45+/CD3+/CD4+ and regulatory T cells as CD45+/CD4+/CD25+/Foxp3+. We used the BD LSRII cytometer at the Centre Imagerie Cellulaire Santé platform in Clermont‐Ferrand. Figure S2 shows the representative dot plots of flow cytometric analysis.

### 2.9 Statistics

Statistical analyses were conducted using Prism software version 6.01 (GraphPad, San Diego, California, USA). *p*‐value < 0.05 was considered to represent statistically significant differences. Results were expressed as mean ± standard deviation and analysed statistically by *t*‐test or one and two‐way ANOVA.

## 3 Results

### 3.1 Characterisation of Tumour Growth and Pigmentation of Three Murine Melanoma Models

**FIGURE 1:**
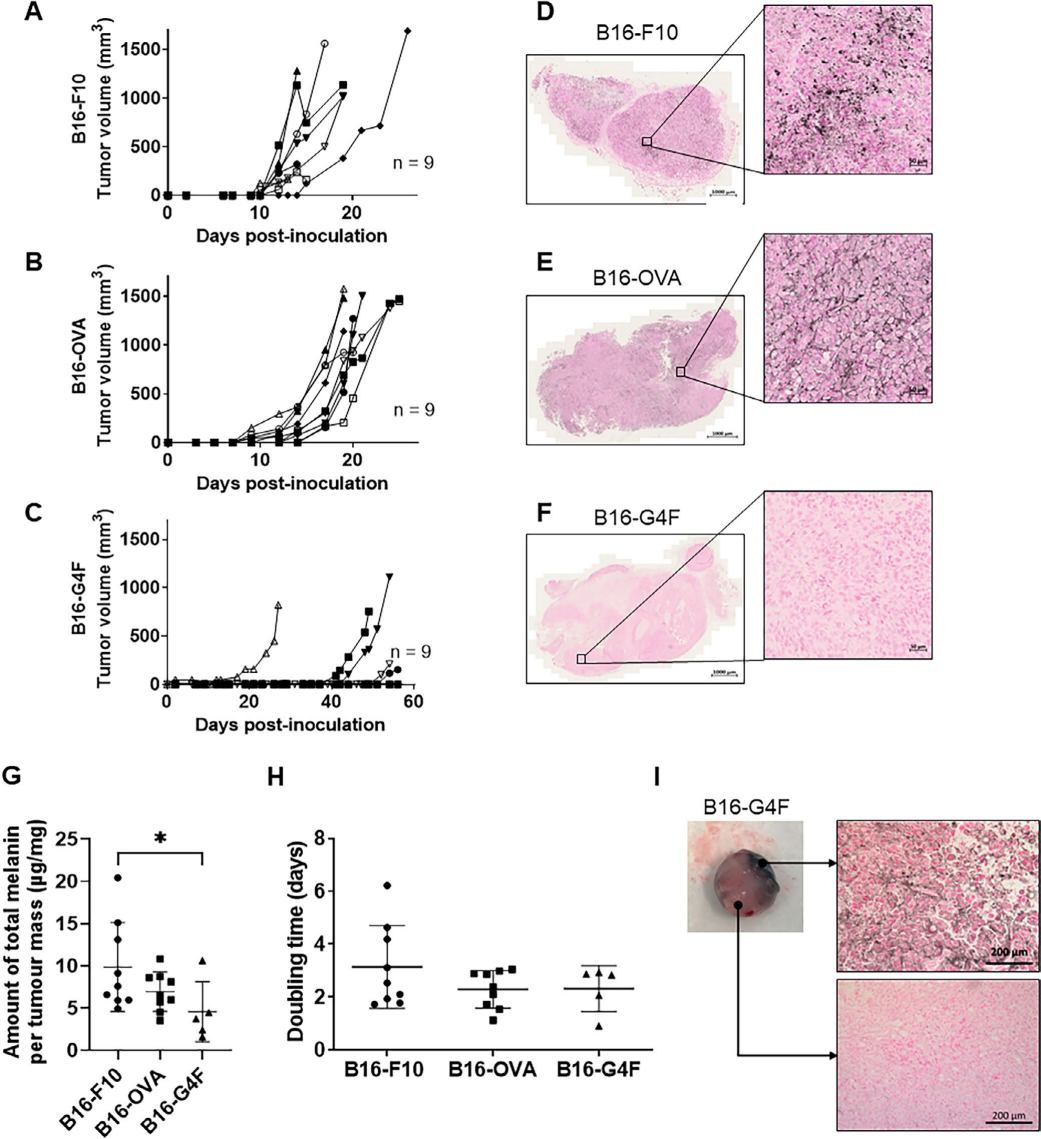
Characterisation of tumour growth and pigmentation of three murine melanoma models. (A–C) C57BL6/J mice (*n* = 9 per group) were injected subcutaneously with 200,000 B16‐F10 cells (A), B16‐OVA cells (B) and B16‐G4F cells (C) into the right flank. D, E and F. Fontana Masson staining highlights pigmentation in B16‐F10 (D), B16‐OVA (E) and B16‐G4F tumours (F) into the right flank. (G) The amount of melanin was quantified in each mouse. Data are expressed as mean ± SD, Multiple *t*‐test, *p* < 0.05 = *; *p* < 0.01 = **; *p* < 0.001 = ***. (H) The DT for each model was calculated. (I) Photography of a repigmented B16‐G4F tumour at 1000 mm^3^ stage, Fontana Masson stains highlighting the pigmented and non‐pigmented sides.

Inoculating 2 × 10^5^ cells reliably induced tumour growth in pigmented B16‐F10 and B16‐OVA murine models, while the non‐pigmented B16‐G4F model showed variability (Figure 1A–C). DT showed no statistical differences between the three models (B16‐F10 = 3.1 ± 1.6 days; B16‐OVA = 2.3 ± 0.7 days; B16‐G4F = 2.3 ± 0.9 days) (Figure 1H). Fontana Masson (Figure 1D–F) and spectrophotometry assay (Figure 1G) revealed pigmentation levels, confirming pigmentation in B16‐F10 and B16‐OVA models but not in B16‐G4F. However, B16‐G4F tumours were observed to regain pigmentation in vivo, confirmed by Fontana Masson staining (Figure 1I).

### 3.2 Characterisation of Immune Microenvironment in the Three Murine Models

**FIGURE 2:**
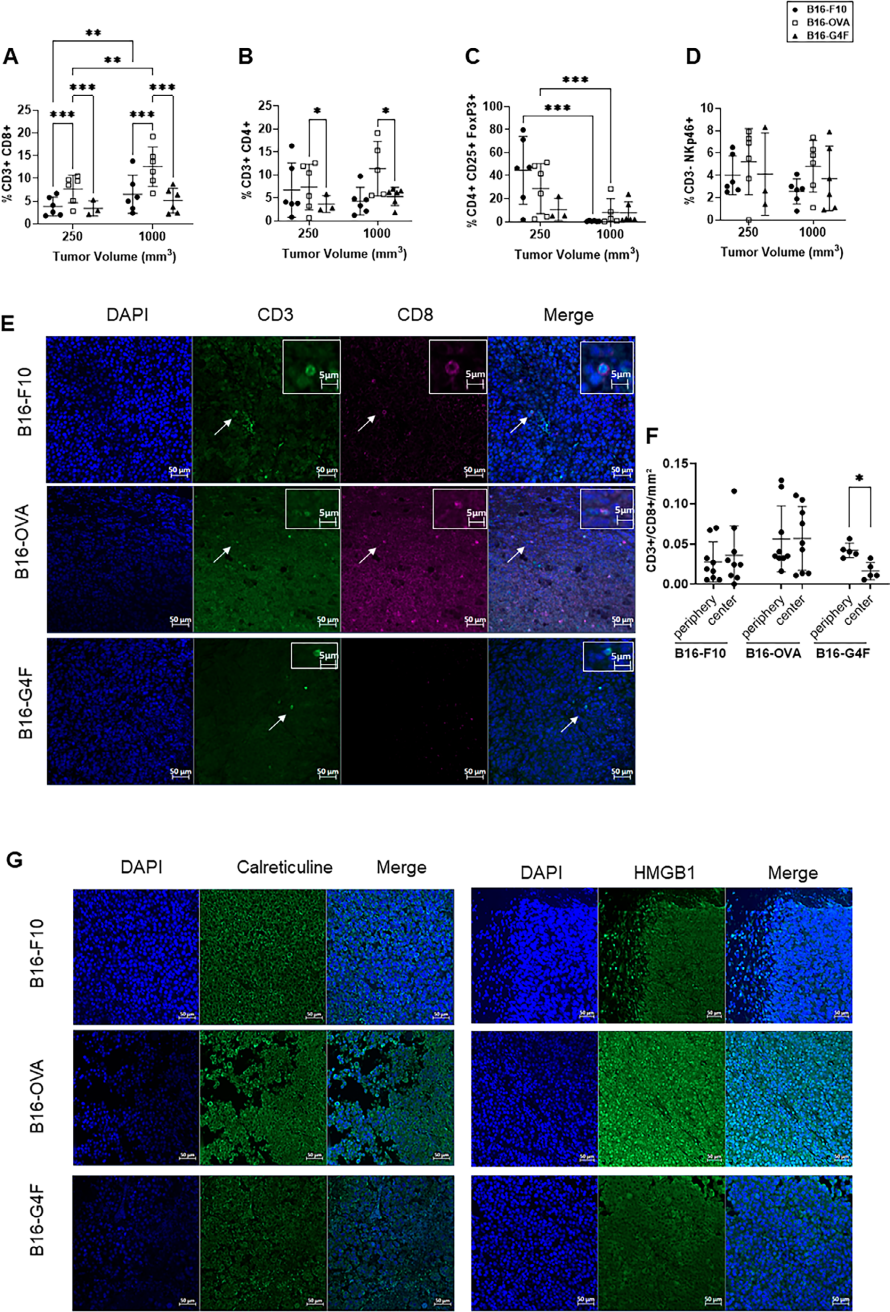
Characterisation of the immune microenvironment in murine models B16‐OVA, B16‐F10 and B16‐G4F. (A–D) Murine models B16‐OVA, B16‐F10 and B16‐G4F were monitored and characterised by flow cytometry at different stages 250 mm^3^ and 1000 mm^3^. Number of tumour infiltrating lymphocytes per gram was quantified by flow cytometry (*n* = 6 per time and condition except for the B16‐G4F model due to its poor tumour uptake). Gating was performed among CD45+ cells. CD8 T cells were identified as CD3+/CD8+ cells (A), CD4 T cells were identified as CD3+/CD4+ cells (B), regulatory T cells were identified as CD4+/CD25+/FoxP3 (C), NK cells were identified as CD3–/NKp46+ (D). (E) CD3 + CD8+ immunofluorescence allows a spatial representation of CD8+ T lymphocytes in the three models. (F) Quantification of CD3^+^/CD8^+^ cells per mm^2^ in different experimental groups (B16‐P10, B16‐OVA, B16‐G4F), comparing periphery and centre tumours. Each dot represents an individual mouse. Horizontal bars indicate mean ± standard deviation. A significant decrease in CD8^+^ cell infiltration is observed between periphery and centre in B16‐G4F tumours (*p < 0.05). (G) Immunofluorescence of calreticulin and HMGB1 provides a basal representation of immunogenic cell death markers. Data are expressed as mean + SD, Two‐way ANOVA and One‐way ANOVA, *p* < 0.05 = *; *p* < 0.01 = **; *p* < 0.001 = ***.

Flow cytometry analysis showed a significantly higher level of cytotoxic CD8+ T cells (Figure 2A, *p* < 0.001) and helper CD4+ T cells (*p* < 0.05) (Figure 2B) infiltration in the B16‐OVA model at both tumour stages (250 and 1000 mm^3^) compared to other models. The CD8+ T cells proportion was also significantly higher at the 1000 mm^3^ stage in B16‐F10 and B16‐OVA tumours. There was also a higher rate of T regulatory cells in the B16‐F10 and B16‐OVA models at the 250 mm^3^ stage than at the 1000 mm^3^ stage (*p* < 0.001) (Figure 2C). However, no difference in the rate of NK cells was observed between models (B16‐0VA vs. B16‐F10 *p* = 0.08; B16‐0VA vs. B16‐G4F *p* = 0.34; B16‐F10 vs. B16‐G4F *p* = 0.51) (Figure 2D). CD8+ T cells showed no spatial distribution difference between the periphery and the center in B16‐F10 and B16‐OVA tumours, while there was a statistical difference in B16‐G4F tumours (*p* < 0.05) (Figure 2E,F and Figure 1S). CLR and HMGB1, markers of DAMPs, were diffusely labelled and appeared more expressed in the B16‐OVA model compared to other models (Figure 2G).

### 3.3 Development of a Non‐Pigmented Melanoma Cell Line

**FIGURE 3:**
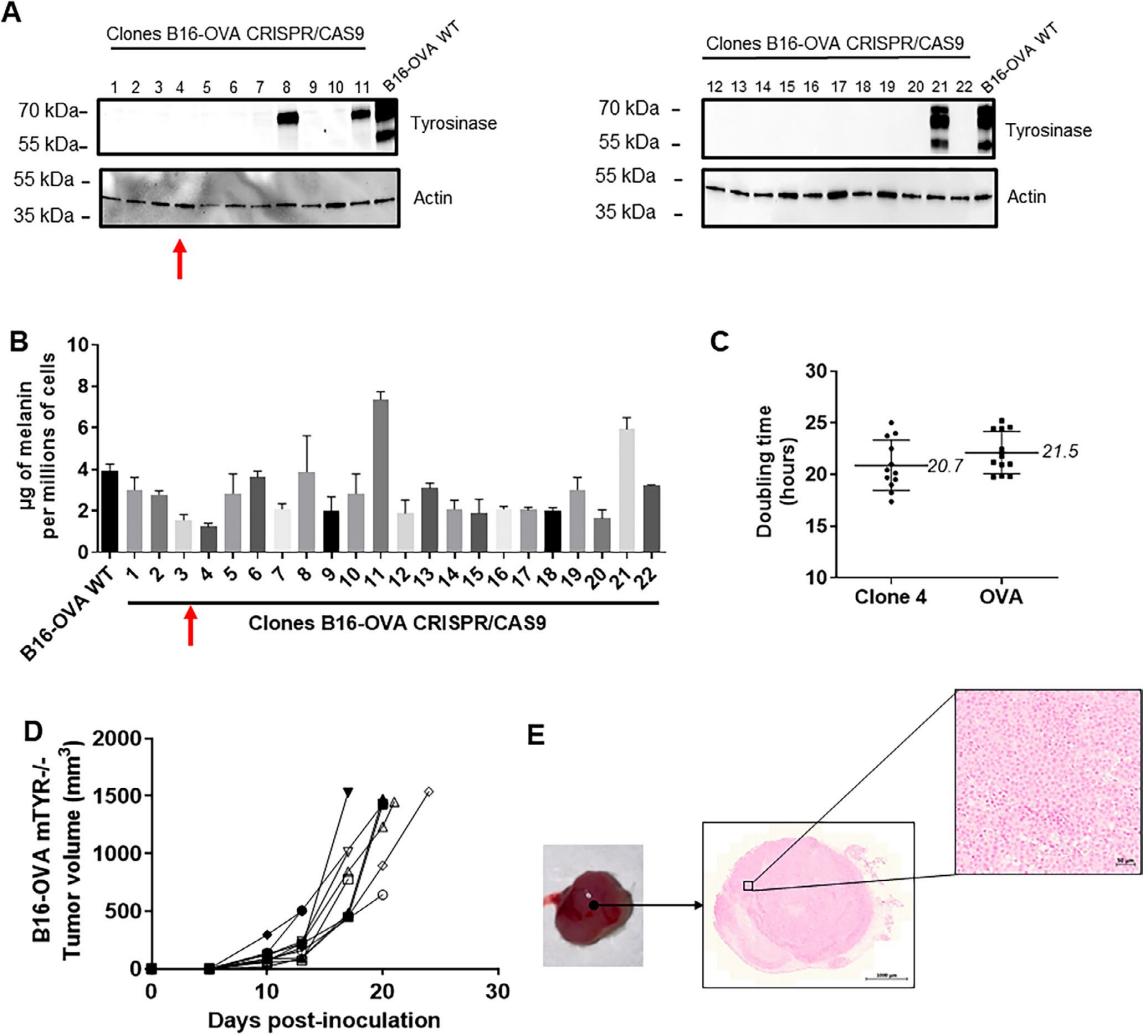
Screening of clones obtained via CRISPR/CAS9 on B16‐OVA cells. (A) Screening of CRISPR‐CAS9 clones B16‐OVA (*n* = 22) by western blot for protein tyrosinase accumulation; red arrow represents clone number 4. (B) The melanin content of each clone was quantified using a spectrophotometric method. (C) Doubling time of clone 4 B16‐OVA mTYR−/− and B16‐OVA cells has been made from time‐lapse (Incucyte). (D) Time‐dependent‐up of C57BL6/J mice inoculated with clone number 4 (*n* = 10). (E) Image showing one B16‐OVA mTYR−/− tumour and the Fontana‐Masson staining (red arrow show clone 4). Two‐way ANOVA and one‐way ANOVA, *p* < 0.05 = *; *p* < 0.01 = **; *p* < 0.001 = ***.

Results of tumour growth and re‐pigmentation in B16‐G4F led us to reconsider this non‐pigmented model. Therefore, we employed CRISPR/Cas9 technology on B16‐OVA cells to invalidate the tyrosinase gene. From this, twenty‐two B16‐OVA clones were generated and verified via western blot, confirming the absence of tyrosinase protein accumulation. All clones exhibited tyrosinase KO, except clones 8, 11 and 21 (Figure 3A). Clone 4 demonstrated the lowest melanin content (1.2 ± 0.2 μg of melanin/millions of cells) (Figure 3B). DT were similar for both cell lines (B16‐OVA WT: 21.5 ± 1.9; clone 4: 20.7 ± 6.0 h) (Figure 3C). Clone 4 was then inoculated into 5‐week‐old female C57BL6/J mice with 500,000 cells. DT values (2.4 ± 0.5 days) were homogeneous in mice inoculated with this new non‐pigmented melanoma cell line, renamed B16‐OVAmTYR−/− (Figure 3D). Remarkably, no re‐pigmentation was observed after macroscopic observation and Fontana Masson staining at stage 1500 mm^3^ (Figure 3E).

### 3.4 Characterisation of a New Double Pigmented and Non‐Pigmented Tumour Model

**FIGURE 4:**
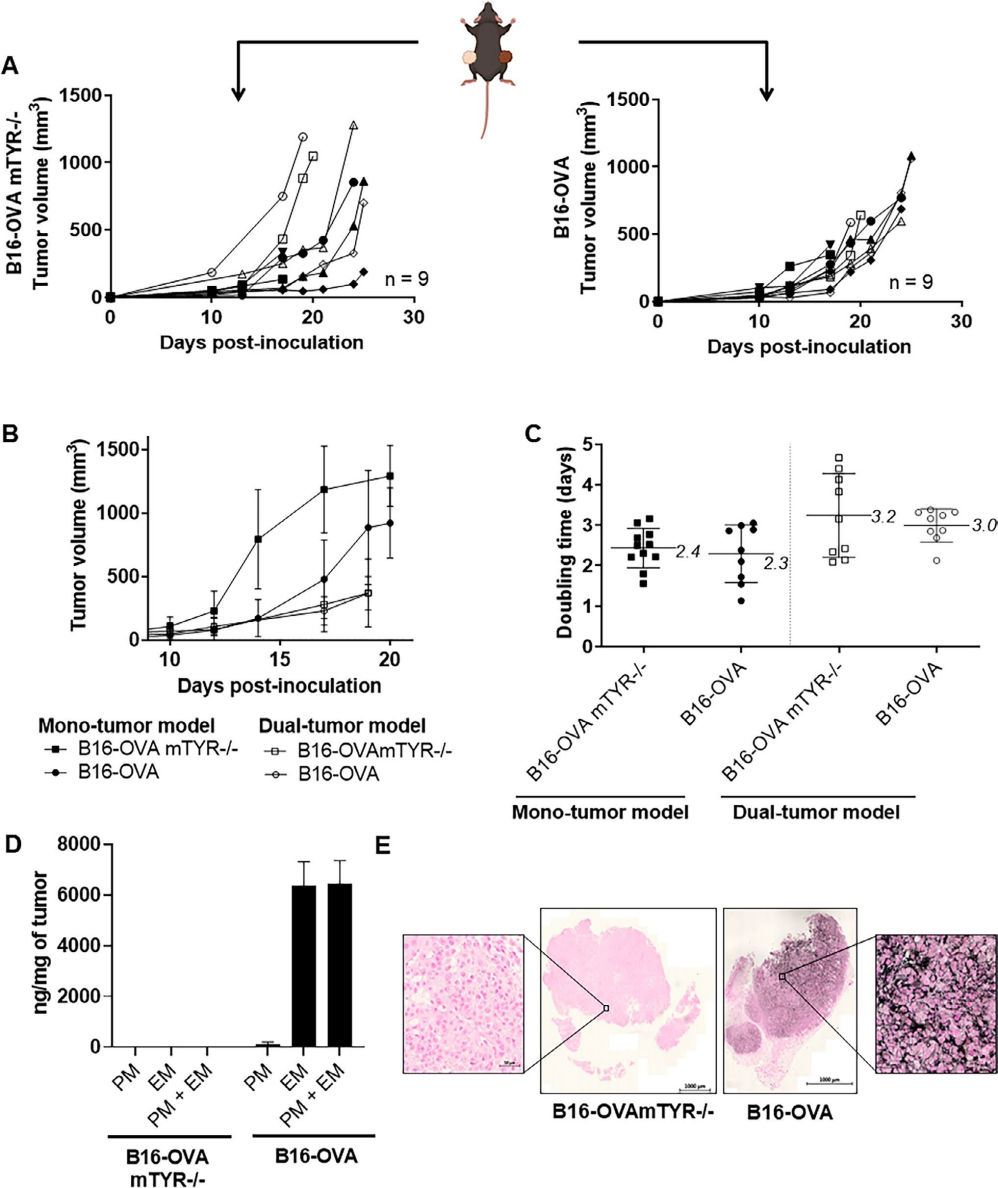
Characterisation of a new double pigmented and non‐pigmented tumour model. (A) Time dependent follow up of double pigmented (B16‐OVA) and non‐pigmented (B16‐OVA mTYR−/−) tumour model (*n* = 9). (B) Average tumour volumes for different OVA models and the doubling time for each OVA model were calculated and noted in brackets. (C) Tumour doubling time in mono‐tumour and dual‐tumour models. Each dot represents an individual tumour, and horizontal bars indicate mean ± SD. Mean doubling times are indicated above each group. (D) Quantification of different melanin types in the two different tumours B16‐OVA mTYR−/− and B16‐OVA (*n* = 3 per tumour). (E) Fontana Masson staining highlights pigmentation in B16‐OVA and B16‐OVA mTYR−/− tumours (scale = 1000 μm; scale = 50 μm).

**FIGURE 5:**
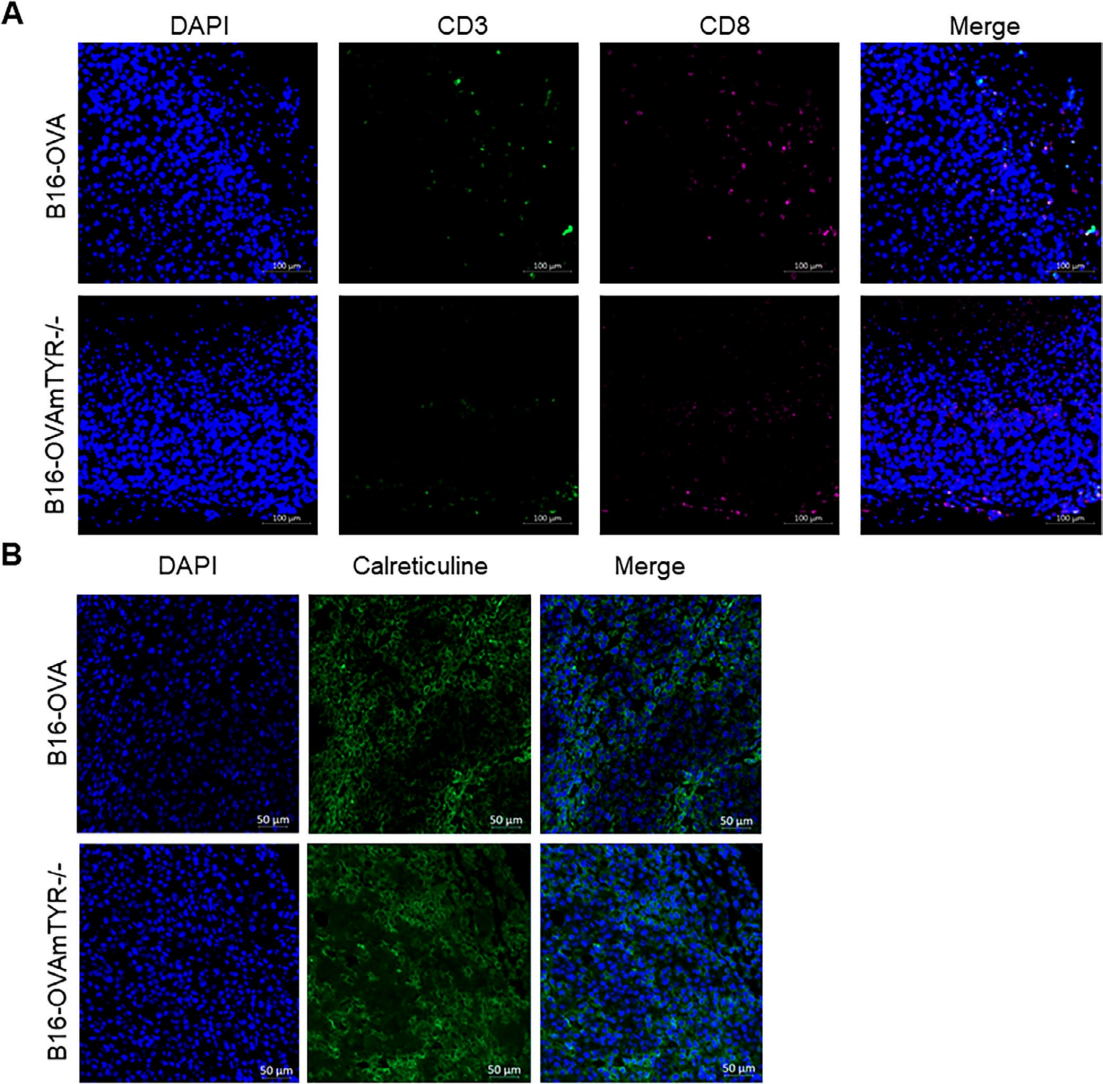
Characterisation of the immune microenvironment in new double‐pigmented and non‐pigmented tumour model. Immunofluorescence of CD3+/CD8+ (A) and calreticulin (B) provides a basal representation of immunogenic cell death markers of double pigmented (B16‐OVA) and non‐pigmented (B16‐OVA mTYR−/−) tumour model.

Double inoculation of B16‐OVAmTYR−/− (non‐pigmented) and B16‐OVA (pigmented) resulted in homogeneous tumour growth on both sides of the mouse flank (Figure 4A). DT of non‐pigmented (3.2 ± 1.0 days) and pigmented (3.0 ± 0.4 days) tumours was unaffected, regardless of the inoculation model (Figure 4B,C). Pigmented tumours showed high EM levels and PM levels, while non‐pigmented tumours showed no detectable pigment (Figure 4D). Fontana Masson staining revealed brown/black markings on pigmented tumours and no staining on non‐pigmented tumours (Figure 4E). No difference in the expression of DAMPs (CD3+/CD8+, calreticulin and HMGB1) was found using immunohistochemistry between the mono‐tumour and double tumour models (Figure 5).

## 4 Discussion

This study presents the development of a novel murine dual‐tumour model, featuring pigmented (B16‐OVA) and non‐pigmented (B16‐OVA mTYR−/−) melanoma, that closely mirrors the clinical coexistence of phenotypically distinct metastatic sites in melanoma patient. This model could be used to assess the value of new therapeutic modalities and, in particular, the ability of the abscopal effect obtained after melanin‐targeted therapies to stimulate co‐treatments such as ICIs, photodynamic therapy [25] or oncolytic viruses [26] as they trigger systemic immune responses. Additionally, this model could allow for the study of the antioxidant power of melanin on the therapeutic response to oxidative stress inducers such as ionising radiation or chemotherapy.

We develop this model to study the abscopal effect induced by our home‐made TRT molecule, [^131^I]ICF01012, a quinoxaline‐derived compound that targets melanin in melanoma. One of the main challenges was to use a murine melanoma with immunogenicity close to human melanoma immune microenvironement [27]. Leick et al. transfected B16 cells with OVA or AAD antigen (AAD is a chimeric class I MHC molecule) and showed that immune‐cell infiltration in murine melanomas varies by tumour type and location (intraperitoneal (i.p). or s.c.). Interestingly, i.p. location was characterised by higher immune‐cell counts in B16‐OVA tumours [28]. Studies also found a similar low density of immune infiltrate in B16‐F10 melanoma and a higher infiltrate in B16‐OVA [29]. Additionally, we note an influence of the size of the tumour on the proportion of CD8+ T cells in B16‐OVA, suggesting that a higher tumour volume releases more tumour antigens and DAMPs, leading to an enhanced cytotoxic lymphocyte recruitment. This parameter should be further considered to determine the day of TRT injection in future experimentations. Moreover, in human melanoma, infiltration of immune cells (i.e., CD8+ T cells, NK or CD4+ helper) into the tumour microenvironment is associated with improved survival and clinical response to immune therapies [30]. All these data confirm that the B16‐OVA model mimics immune cell populations found in high proportions in human melanoma. In addition, B16‐OVA appears to spontaneously translocate CALR to the membrane and release HMGB1, markers of ICD, confirming the high immunogenicity of this cell line.

The second challenge was to use a non‐pigmented murine melanoma with similar growth and immunogenicity characteristics to the selected pigmented model (B16‐OVA). To develop this new murine model, we first compared pigmented (B16‐F10, B16‐OVA) and non‐pigmented (B16‐G4F) melanoma cell lines. B16‐F10 and B16‐OVA showed consistent pigmentation and tumour growth. B16‐G4F, lacking MC1R receptors, was initially considered to be a non‐pigmented model [19]. There was no existing in vivo data on the B16‐G4F cell line in the literature. When inoculated in C57BL6/J mice, B16‐G4F cells showed heterogeneous growth and re‐pigmentation, making them unsuitable for studying the abscopal effect. This phenomenon mirrors clinical observations where some amelanotic metastases regain melanin production ability [31]. Re‐pigmentation of amelanotic tumours can occur due to gene expression changes such as mutations in the MITF gene [32] or changes in the tumour microenvironment [33]. Skoniecka et al. showed that amelanotic melanoma cells can become melanized when grown in media rich in specific amino acids [33]. Given the inadequacy of the B16‐G4F model in terms of pigmentation, we have engineered a B16‐OVA KO strain targeting the tyrosinase gene. This approach enabled the establishment of a B16‐OVA derived cell line exhibiting no pigmentation while maintaining an identical genetic background to B16‐OVA wild‐type. This clone, when inoculated in mice, showed homogeneous tumour growth, no melanin content, and no re‐pigmentation. These mono‐tumour models could serve as negative controls to evaluate a potential abscopal effect.

Simultaneously inoculating both B16‐OVA and B16‐OVA mTYR−/− cell lines showed similar DT on both sides of the mouse flank. Non‐pigmented tumours did not contain any melanin (PM or EM), and there were no significant differences in DT between single‐tumour models and the dual‐tumour model. Notably, this simultaneous inoculation slightly slowed tumour growth compared to the mono‐tumour model. Furthermore, it is interesting to note that double‐tumour murine models of melanoma exist in the literature, for example with two B16‐F10 tumours [34] or two B16‐OVA tumours in C57BL/6 [35], but none exist with one pigmented tumour and one non‐pigmented tumour. This type of model is recommended in the guidelines for the study of ICD and the abscopal effect [36], which recommend generating two s.c. lesions on distant sites using cancer cells in immunocompetent mice. We developed a similar metastatic model with two palpable s.c. tumours, one pigmented and the other non‐pigmented, to assess potential tumour regression post‐treatment. Another approach could have been injecting pigmented cells s.c. and non‐pigmented cells intravenously to induce lung colonies, similar to the previously described B16BL6 melanoma model [37]. Our new dual pigmented/non‐pigmented tumour model follows the guidelines proposed by Galluzi et al. [38]. This model serves as a proof of concept for the feasibility of developing a dual‐tumour model where one tumour expresses a target of interest and the other does not, achieved through genetic modulation techniques. The loss or overexpression of certain targets is a common phenomenon in oncology [39], suggesting that this methodology for developing a murine model could be extrapolated to other types of cancer.

There is growing interest in combining ionising radiation with immunotherapy, Saiag et al. demonstrated that external hypofractionated radiotherapy combined with anti‐PD‐1 therapy produced encouraging results in melanoma patients who had not responded to anti‐PD‐1 therapy alone [40, 41, 42]. This combination also demonstrated a potential abscopal effect, where non‐irradiated tumours regressed due to specific immune response [40, 41, 42] induced by external radiotherapy. The benefits of TRT include targeting tumour cells with greater precision and minimising damage to surrounding healthy tissue [43]. Consequently, it would be interesting to combine ICI with TRT in order to increase their efficacy through the possible abscopal effect of TRT [44] in this model. Data corroborate that the high immunogenicity of the B16‐OVA model is a valid model for studying response to ICI in combination with TRT [45]. Combining these three factors (B16‐OVA model, abscopal effect and immunotherapy) opens up new prospects for research improving cancer treatment [12, 43, 46].

To conclude, our newly developed dual pigmented and non‐pigmented melanoma mouse model faithfully mimics a clinical scenario, facilitating a detailed exploration of the abscopal effect in metastatic melanoma. This model is driving our current research on the abscopal effect post‐TRT in metastatic melanoma, serving as an innovative proof of concept by deleting a specific molecular target.

## Author Contributions

Data aquisition: M.D., B.C., N.H., S.B., J.D., M.Q. Conception and design: M.D., E.J., J.R., S.B., C.M., P.R., M.D.I. Data analysis and interpretation: M.D., A.D., P.O.R., C.M., E.M., E.M.‐N., M.Q., M.D.I., E.C., E.J., J.R. All authors have read and approved the final manuscript.

## Ethics Statement

Mice experiments were approved by local Ethic committee of Clermont‐Ferrand and French Ministry of Education and Research (Approval No. 2022050215457597).

## Conflicts of Interest

The authors declare no conflicts of interest.

## Supporting information


**Figure S1:** Negative controls for immunofluorescence. CD3^+^/CD8^+^ (A), calreticulin (B), and HMGB1 (C) staining, providing baseline representation of immunogenic cell death markers in the three murine models.

**Figure S2:** Representative dot‐plots for each type of tumour: B16F10 tumour (A), B16‐OVA (B) and B16‐G4F (C). Gating was carried out among CD45+ cells. NK cells were identified as CD3‐/NKp46+, CD8+ T cells as CD45+/CD3+/CD8+, CD4+ T cells as CD45+/CD3+/CD4+ and regulatory T cells as CD45+/CD4+/CD25+/Foxp3 + .

## Data Availability

The data that support the findings of this study are available from the corresponding author upon reasonable request.

## References

[1] S. M. Ostrowski and D. E. Fisher , “Biology of Melanoma,” Hematology/Oncology Clinics of North America 35 (2021): 29–56. 33759772 10.1016/j.hoc.2020.08.010

[2] R. M. Slominski , M. A. Zmijewski , and A. T. Slominski , “The Role of Melanin Pigment in Melanoma,” Experimental Dermatology 24 (2015): 258–259. 25496715 10.1111/exd.12618 PMC4450257

[3] K. Saginala , A. Barsouk , J. S. Aluru , P. Rawla , and A. Barsouk , “Epidemiology of Melanoma,” Medical Science 9 (2021): 63. 10.3390/medsci9040063 PMC8544364 34698235

[4] Z. R. Garrison , C. M. Hall , R. M. Fey , et al., “Advances in Early Detection of Melanoma and the Future of At‐Home Testing,” Life (Basel) 13 (2023): 974. 37109503 10.3390/life13040974 PMC10145469

[5] R. W. Jenkins and D. E. Fisher , “Treatment of Advanced Melanoma in 2020 and Beyond,” Journal of Investigative Dermatology 141 (2021): 23–31. 32268150 10.1016/j.jid.2020.03.943 PMC7541692

[6] G. V. Long , D. Stroyakovskiy , H. Gogas , et al., “Dabrafenib and Trametinib Versus Dabrafenib and Placebo for Val600 BRAF‐Mutant Melanoma: A Multicentre, Double‐Blind, Phase 3 Randomised Controlled Trial,” Lancet 386 (2015): 444–451. 26037941 10.1016/S0140-6736(15)60898-4

[7] J. Larkin , V. Chiarion‐Sileni , R. Gonzalez , et al., “Five‐Year Survival With Combined Nivolumab and Ipilimumab in Advanced Melanoma,” New England Journal of Medicine 381 (2019): 1535–1546. 31562797 10.1056/NEJMoa1910836

[8] P. Savoia , E. Zavattaro , and O. Cremona , “Clinical Implications of Acquired BRAF Inhibitors Resistance in Melanoma,” International Journal of Molecular Sciences 21 (2020): 9730. 33419275 10.3390/ijms21249730 PMC7766699

[9] S. Y. Lim , E. Shklovskaya , J. H. Lee , et al., “The Molecular and Functional Landscape of Resistance to Immune Checkpoint Blockade in Melanoma,” Nature Communications 14 (2023): 1516. 10.1038/s41467-023-36979-y PMC10024679 36934113

[10] R. H. Mole , “Whole Body Irradiation—Radiobiology or Medicine?,” British Journal of Radiology 26 (1953): 234–241. 13042090 10.1259/0007-1285-26-305-234

[11] E. B. Golden and L. Apetoh , “Radiotherapy and Immunogenic Cell Death,” Seminars in Radiation Oncology 25 (2015): 11–17. 25481261 10.1016/j.semradonc.2014.07.005

[12] M. Widel , “Radionuclides in Radiation‐Induced Bystander Effect; May It Share in Radionuclide Therapy?,” Neoplasma 64 (2017): 641–654. 28592116 10.4149/neo_2017_501

[13] W. Echavidre , D. Fagret , M. Faraggi , V. Picco , and C. Montemagno , “Recent Pre‐Clinical Advancements in Nuclear Medicine: Pioneering the Path to a Limitless Future,” Cancers 15 (2023): 4839. 37835533 10.3390/cancers15194839 PMC10572076

[14] K. J. McKelvey , A. L. Hudson , M. Back , T. Eade , and C. I. Diakos , “Radiation, Inflammation and the Immune Response in Cancer,” Mammalian Genome 29 (2018): 843–865. 30178305 10.1007/s00335-018-9777-0 PMC6267675

[15] S. C. Kleinendorst , E. Oosterwijk , J. Bussink , H. Westdorp , M. W. Konijnenberg , and S. Heskamp , “Combining Targeted Radionuclide Therapy and Immune Checkpoint Inhibition for Cancer Treatment,” Clinical Cancer Research 28 (2022): 3652–3657. 35471557 10.1158/1078-0432.CCR-21-4332 PMC9433955

[16] J. Rouanet , V. Benboubker , H. Akil , et al., “Immune Checkpoint Inhibitors Reverse Tolerogenic Mechanisms Induced by Melanoma Targeted Radionuclide Therapy,” Cancer Immunology, Immunotherapy 69 (2020): 2075–2088. 32447411 10.1007/s00262-020-02606-8 PMC11027634

[17] P. A. Clark , R. N. Sriramaneni , A. M. Bates , et al., “Low‐Dose Radiation Potentiates the Propagation of Anti‐Tumor Immunity Against Melanoma Tumor in the Brain After In Situ Vaccination at a Tumor Outside the Brain,” Radiation Research 195 (2021): 522–540. 33826741 10.1667/RADE-20-00237.1 PMC8259713

[18] J. Choi , W. Beaino , R. J. Fecek , et al., “Combined VLA‐4‐Targeted Radionuclide Therapy and Immunotherapy in a Mouse Model of Melanoma,” Journal of Nuclear Medicine 59 (2018): 1843–1849. 29959213 10.2967/jnumed.118.209510 PMC6278902

[19] F. F. Solca , J. C. Tapia , K. Iwata , and A. N. Eberle , “B16‐G4F Mouse Melanoma Cells: An MSH Receptor‐Deficient Cell Clone,” FEBS Letters 322 (1993): 177–180. 8482388 10.1016/0014-5793(93)81563-f

[20] F. A. Ran , P. D. Hsu , J. Wright , V. Agarwala , D. A. Scott , and F. Zhang , “Genome Engineering Using the CRISPR‐Cas9 System,” Nature Protocols 8 (2013): 2281–2308. 24157548 10.1038/nprot.2013.143 PMC3969860

[21] K. Labun , T. G. Montague , M. Krause , Y. N. Torres Cleuren , H. Tjeldnes , and E. Valen , “CHOPCHOP v3: Expanding the CRISPR Web Toolbox Beyond Genome Editing,” Nucleic Acids Research 47 (2019): W171–W174. 31106371 10.1093/nar/gkz365 PMC6602426

[22] M. Bonnet , F. Mishellany , J. Papon , et al., “Anti‐Melanoma Efficacy of Internal Radionuclide Therapy in Relation to Melanin Target Distribution,” Pigment Cell & Melanoma Research 23 (2010): e1–e11. 10.1111/j.1755-148X.2010.00716.x 20444199

[23] B. Rioux , J. Rouanet , H. Akil , et al., “Determination of Eumelanin and Pheomelanin in Melanomas Using Solid‐Phase Extraction and High Performance Liquid Chromatography‐Diode Array Detection (HPLC‐DAD) Analysis,” Journal of Chromatography. B, Analytical Technologies in the Biomedical and Life Sciences 1113 (2019): 60–68. 30897406 10.1016/j.jchromb.2019.03.010

[24] H. Akil , M. Quintana , J. H. Raymond , et al., “Efficacy of Targeted Radionuclide Therapy Using [131I]ICF01012 in 3D Pigmented BRAF‐ and NRAS‐Mutant Melanoma Models and In Vivo NRAS‐Mutant Melanoma,” Cancers 13 (2021): 1421. 33804655 10.3390/cancers13061421 PMC8003594

[25] S. Ali Mohammad , A. Hak , S. V. Pogu , and A. K. Rengan , “Radiotherapy, Photodynamic Therapy, and Cryoablation‐Induced Abscopal Effect: Challenges and Future Prospects,” Cancer Innovation 2 (2023): 323–345. 38090387 10.1002/cai2.53 PMC10686191

[26] P. K. Bommareddy , M. Shettigar , and H. L. Kaufman , “Integrating Oncolytic Viruses in Combination Cancer Immunotherapy,” Nature Reviews. Immunology 18 (2018): 498–513. 10.1038/s41577-018-0014-6 29743717

[27] A. Passarelli , F. Mannavola , L. S. Stucci , M. Tucci , and F. Silvestris , “Immune System and Melanoma Biology: A Balance Between Immunosurveillance and Immune Escape,” Oncotarget 8 (2017): 106132–106142. 29285320 10.18632/oncotarget.22190 PMC5739707

[28] K. M. Leick , J. Pinczewski , I. S. Mauldin , et al., “Patterns of Immune Cell Infiltration in Murine Models of Melanoma: Roles of Antigen and Tissue Site in Creating Inflamed Tumors,” Cancer Immunology, Immunotherapy 68 (2019): 1121–1132. 31134297 10.1007/s00262-019-02345-5 PMC6887106

[29] J. D. Peske , E. D. Thompson , L. Gemta , R. A. Baylis , Y. X. Fu , and V. H. Engelhard , “Effector Lymphocyte‐Induced Lymph Node‐Like Vasculature Enables Naive T‐Cell Entry Into Tumours and Enhanced Anti‐Tumour Immunity,” Nature Communications 6 (2015): 7114. 10.1038/ncomms8114 PMC4435831 25968334

[30] J. Tian and C. Quek , “Understanding the Tumor Microenvironment in Melanoma Patients With In‐Transit Metastases and Its Impacts on Immune Checkpoint Immunotherapy Responses,” International Journal of Molecular Sciences 25 (2024): 4243. 38673829 10.3390/ijms25084243 PMC11050678

[31] M. C. Botha and B. Lennox , “Re‐Pigmentation of Amelanotic Metastases of Malignant Melanoma by Contact With Epidermis,” Journal of Pathology and Bacteriology 67 (1954): 99–104. 13152622 10.1002/path.1700670112

[32] C. Levy , M. Khaled , and D. E. Fisher , “MITF: Master Regulator of Melanocyte Development and Melanoma Oncogene,” Trends in Molecular Medicine 12 (2006): 406–414. 16899407 10.1016/j.molmed.2006.07.008

[33] A. Skoniecka , M. Cichorek , A. Tyminska , I. Pelikant‐Malecka , and J. Dziewiatkowski , “Melanization as Unfavorable Factor in Amelanotic Melanoma Cell Biology,” Protoplasma 258 (2021): 935–948. 33506271 10.1007/s00709-021-01613-5 PMC8433105

[34] G. Vijayakumar , P. Palese , and P. H. Goff , “Oncolytic Newcastle Disease Virus Expressing a Checkpoint Inhibitor as a Radioenhancing Agent for Murine Melanoma,” eBioMedicine 49 (2019): 96–105. 31676387 10.1016/j.ebiom.2019.10.032 PMC6945240

[35] M. E. Rodriguez‐Ruiz , I. Rodriguez , S. Garasa , et al., “Abscopal Effects of Radiotherapy Are Enhanced by Combined Immunostimulatory mAbs and Are Dependent on CD8 T Cells and Crosspriming,” Cancer Research 76 (2016): 5994–6005. 27550452 10.1158/0008-5472.CAN-16-0549

[36] L. Galluzzi , A. Buqué , O. Kepp , L. Zitvogel , and G. Kroemer , “Immunogenic Cell Death in Cancer and Infectious Disease,” Nature Reviews. Immunology 17 (2017): 97–111. 10.1038/nri.2016.107 27748397

[37] H. Akil , J. Rouanet , C. Viallard , et al., “Targeted Radionuclide Therapy Decreases Melanoma Lung Invasion by Modifying Epithelial‐Mesenchymal Transition‐Like Mechanisms,” Translational Oncology 12 (2019): 1442–1452. 31421458 10.1016/j.tranon.2019.07.015 PMC6704444

[38] L. Galluzzi , I. Vitale , S. Warren , et al., “Consensus Guidelines for the Definition, Detection and Interpretation of Immunogenic Cell Death,” Journal for Immunotherapy of Cancer 8 (2020): e000337. 32209603 10.1136/jitc-2019-000337 PMC7064135

[39] D. Hanahan , “Hallmarks of Cancer: New Dimensions,” Cancer Discovery 12 (2022): 31–46. 35022204 10.1158/2159-8290.CD-21-1059

[40] A. Roger , A. Finet , B. Boru , et al., “Efficacy of Combined Hypo‐Fractionated Radiotherapy and Anti‐PD‐1 Monotherapy in Difficult‐To‐Treat Advanced Melanoma Patients,” Oncoimmunology 7 (2018): e1442166. 30034949 10.1080/2162402X.2018.1442166 PMC6053300

[41] E. Funck‐Brentano , B. Baghad , M. Fort , et al., “Efficacy of Late Concurrent Hypofractionated Radiotherapy in Advanced Melanoma Patients Failing Anti‐PD‐1 Monotherapy,” International Journal of Cancer 147 (2020): 1707–1714. 32083739 10.1002/ijc.32934

[42] P. Saiag , R. Molinier , A. Roger , et al., “Efficacy of Large Use of Combined Hypofractionated Radiotherapy in a Cohort of Anti‐PD‐1 Monotherapy‐Treated Melanoma Patients,” Cancers 14 (2022): 4069. 36077606 10.3390/cancers14174069 PMC9454723

[43] D. J. Craig , N. S. Nanavaty , M. Devanaboyina , et al., “The Abscopal Effect of Radiation Therapy,” Future Oncology 17 (2021): 1683–1694. 33726502 10.2217/fon-2020-0994

[44] K. Reynders , T. Illidge , S. Siva , J. Y. Chang , and D. De Ruysscher , “The Abscopal Effect of Local Radiotherapy: Using Immunotherapy to Make a Rare Event Clinically Relevant,” Cancer Treatment Reviews 41 (2015): 503–510. 25872878 10.1016/j.ctrv.2015.03.011 PMC4816218

[45] J. H. Rowe , I. Elia , O. Shahid , et al., “Formate Supplementation Enhances Antitumor CD8+ T‐Cell Fitness and Efficacy of PD‐1 Blockade,” Cancer Discovery 13 (2023): 2566–2583. 37728660 10.1158/2159-8290.CD-22-1301 PMC10843486

[46] J.‐P. Pouget , A. G. Georgakilas , and J.‐L. Ravanat , “Targeted and Off‐Target (Bystander and Abscopal) Effects of Radiation Therapy: Redox Mechanisms and Risk/Benefit Analysis,” Antioxidants & Redox Signaling 29 (2018): 1447–1487. 29350049 10.1089/ars.2017.7267 PMC6199630

