# Supplementary figures and images for: Targeting Melanin Heterogeneity in Metastatic Melanoma: A Dual‐Tumour Mouse Melanoma Model
Source: Exp Dermatol. 2025 Sep 2;34(9):e70159. doi: 10.1111/exd.70159 (PMC12405743; doi:10.1111/exd.70159)

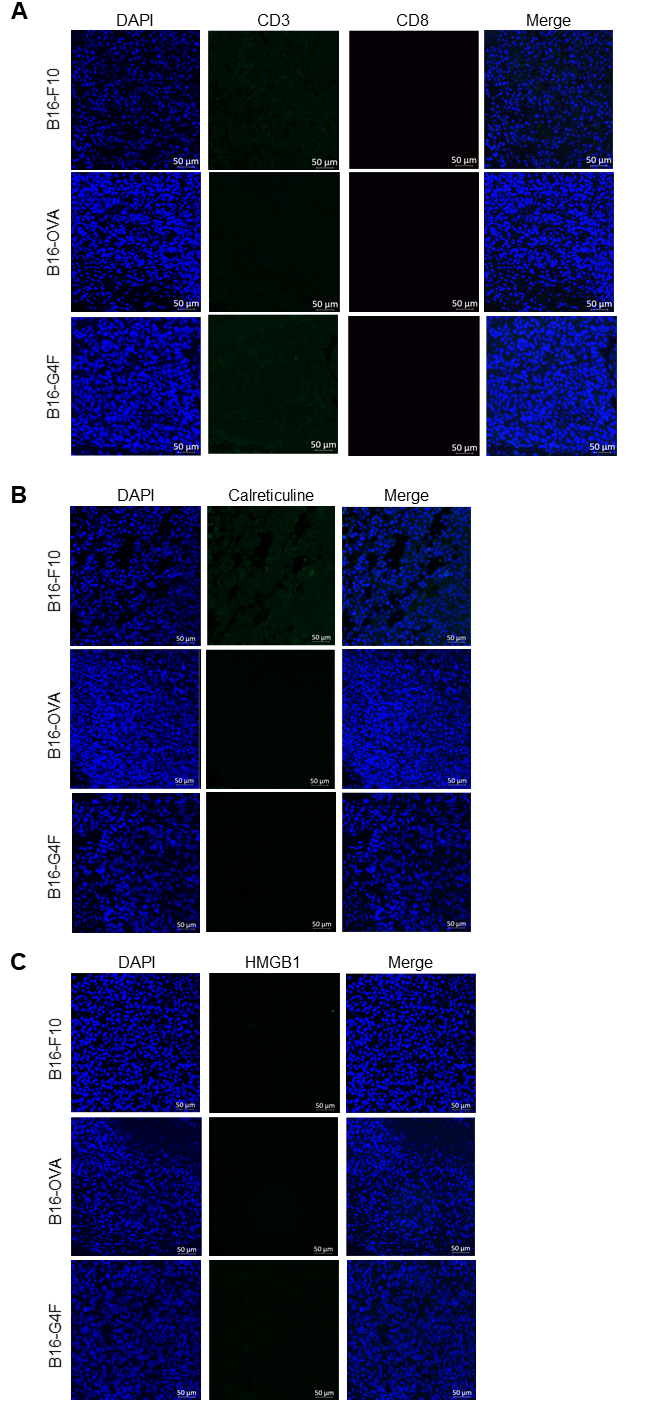

Supplement: Supplementary file 1 — Figure S1: Negative controls for immunofluorescence. CD3+/CD8+ (A), calreticulin (B), and HMGB1 (C) staining, providing baseline representation of immunogenic cell death markers in the three murine models. [file EXD-34-e70159-s002.tif]

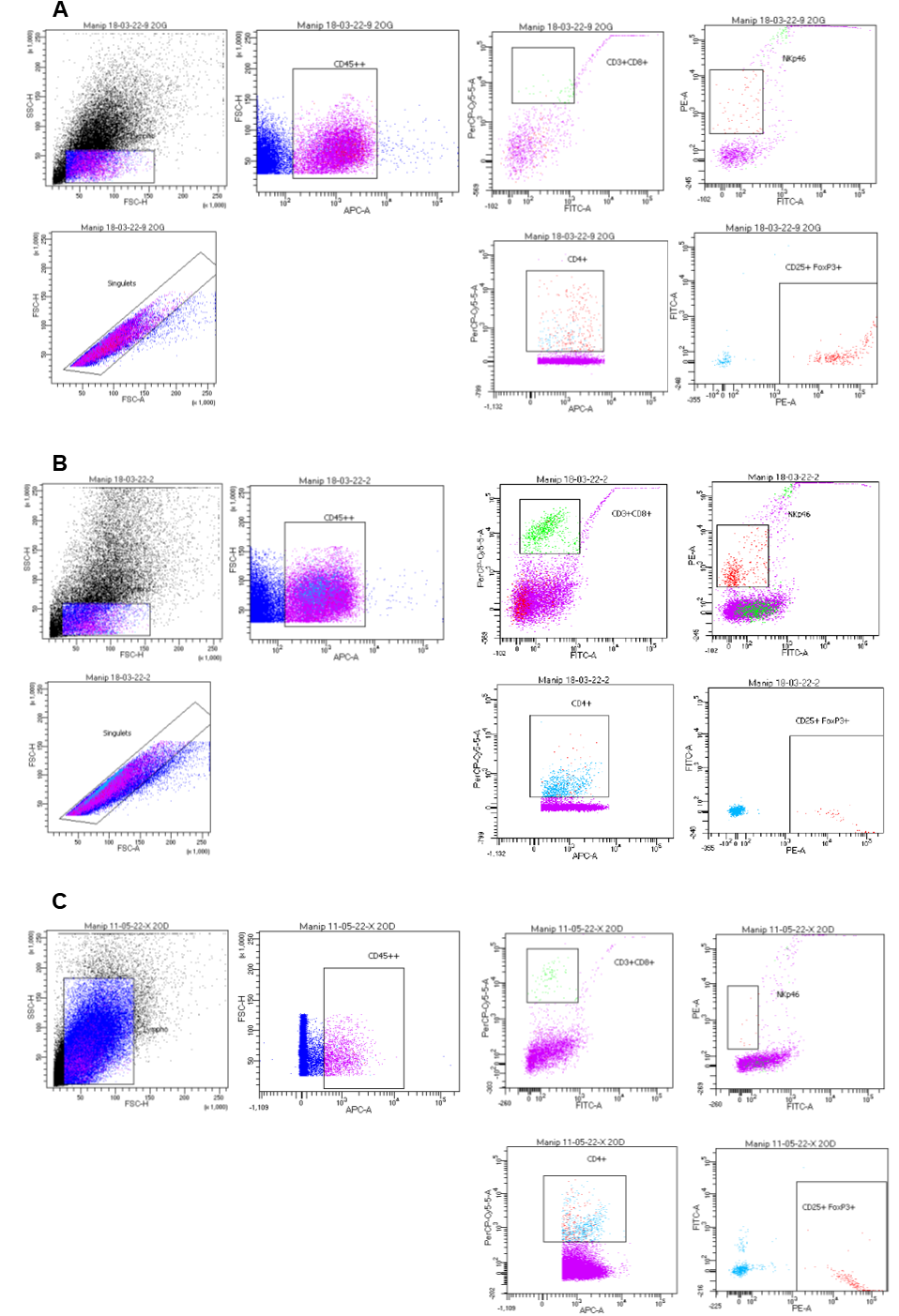

Supplement: Supplementary file 2 — Figure S2: Representative dot‐plots for each type of tumour: B16F10 tumour (A), B16‐OVA (B) and B16‐G4F (C). Gating was carried out among CD45+ cells. NK cells were identified as CD3‐/NKp46+, CD8+ T cells as CD45+/CD3+/CD8+, CD4+ T cells as CD45+/CD3+/CD4+ and regulatory T cells as CD45+/CD4+/CD25+/Foxp3 + . [file EXD-34-e70159-s001.tif]
